# Cocktail of chemical compounds robustly promoting cell reprogramming protects liver against acute injury

**DOI:** 10.1007/s13238-017-0373-y

**Published:** 2017-02-11

**Authors:** Yuewen Tang, Lin Cheng

**Affiliations:** State Key Laboratory of Medical Genomics, Shanghai Institute of Hematology, Rui Jin Hospital, Shanghai Jiao Tong University School of Medicine, Shanghai, 200025 China

**Keywords:** cell reprogramming, chemical compounds, liver regeneration

## Abstract

Tissue damage induces cells into reprogramming-like cellular state, which contributes to tissue regeneration. However, whether factors promoting the cell reprogramming favor tissue regeneration remains elusive. Here we identified combination of small chemical compounds including drug cocktails robustly promoting *in vitro* cell reprogramming. We then administrated the drug cocktails to mice with acute liver injuries induced by partial hepatectomy or toxic treatment. Our results demonstrated that the drug cocktails which promoted cell reprogramming *in vitro* improved liver regeneration and hepatic function *in vivo* after acute injuries. The underlying mechanism could be that expression of pluripotent genes activated after injury is further upregulated by drug cocktails. Thus our study offers proof-of-concept evidence that cocktail of clinical compounds improving cell reprogramming favors tissue recovery after acute damages, which is an attractive strategy for regenerative purpose.

**Electronic supplementary material:**

The online version of this article (doi:10.1007/s13238-017-0373-y) contains supplementary material, which is available to authorized users.

## Introduction

Tissue damage induces cells into reprogramming-like cellular state, which contributes to tissue regeneration (Jessen et al., 2015). Furthermore, genes related with cell reprogramming are activated and play important role during the regeneration process after damage (Bhave et al., 2011). It’s also known that these genes involved are key factors to reprogramming cells into pluripotency *in vitro* and *in vivo* (Mosteiro et al., 2016). Thus it is very interesting to know whether factors promoting cell reprogramming, which are identified through *in vitro* assays, would help tissue regeneration *in vivo*. To date, reports already reveal that cell reprogramming efficiency can be enhanced by chemical compounds, even reprogramming factors can be partially or completely replaced by combination of small chemical compounds in pluripotent cell generation (Huangfu et al., 2008; Ichida et al., 2009; Esteban et al., 2010; Hou et al., 2013). In this paper we explore whether combination of chemical compounds, strongly promoting cell reprogramming but not generating pluripotent cells by themselves alone, would show beneficial effects for mouse liver regeneration after acute injuries.

## Results

### Combination of chemical compounds promote cell reprogramming *in vitro*

**Figure 1:**
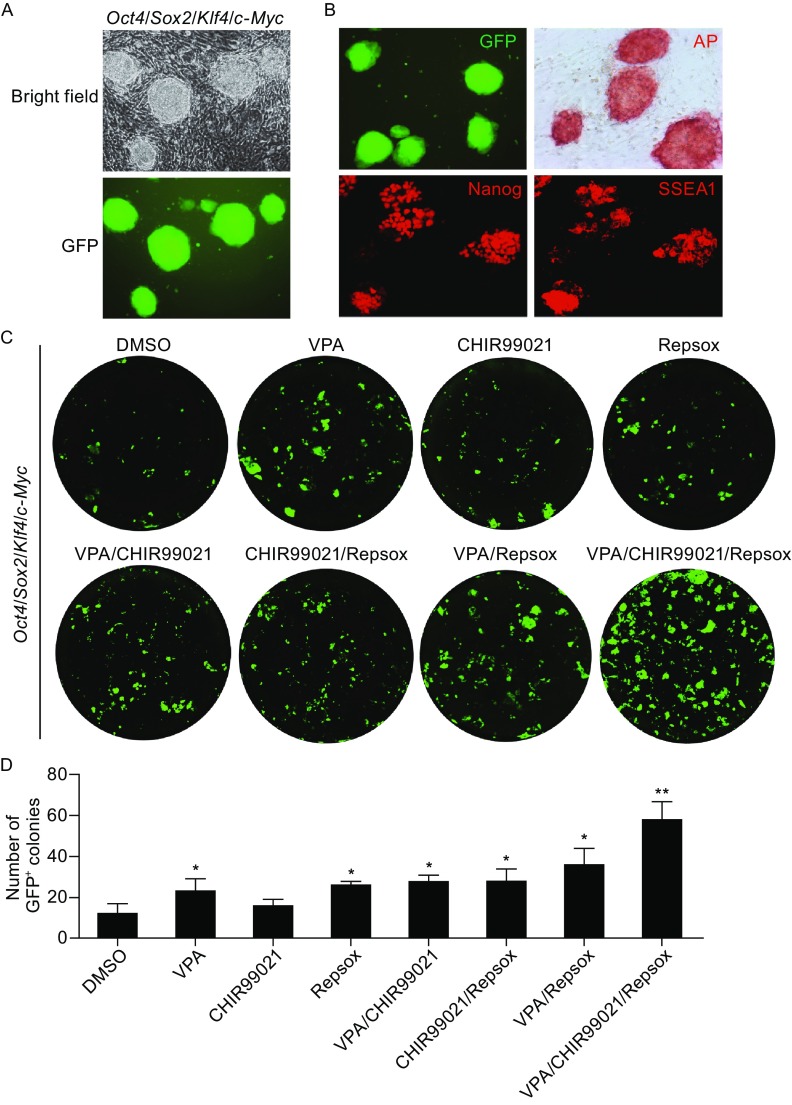
Chemical compounds promote cell reprogramming *in vitro*. (A) Representative images of typical GFP^+^ colonies generated from OG2-MEFs at day 10 post-infection of retroviruses expressing *Oct4*, *Sox2*, *Klf4*, and *c-Myc*. (B) Characterization of Yamanaka factors-induced pluripotent stem cell colonies from OG2-MEFs with alkaline phosphatase (AP) staining and immunofluorescent staining of pluripotent markers Nanog (red) and SSEA1 (red). (C) Generation of pluripotent stem cell from Yamanaka factors-induced OG2-MEFs under treatment of diverse chemical compounds. Images of GFP^+^ colonies were taken on day 10 post-infection. VPA, 0.5 mmol/L. CHIR99021, 3 μmol/L. Repsox, 1 μmol/L. (D) Quantification of GFP^+^ colonies in (C). All figures are representative of three independent experiments (*n* = 3). All data are presented as mean ± SD. *P < 0.05, **P < 0.01 vs. DMSO

To identify small chemical compounds strongly enhancing cell reprogramming efficiency, we set up 96-well-plate-based chemical screening platform for Yamanaka factors-induced reprogramming from mouse embryonic fibroblasts (MEFs). Primary MEFs were derived from OG2^+/−^/ROSA26^+/−^ (OG2) mice (containing a transgenic Oct4-GFP reporter) then transduced with retroviral vectors expressing *Oct4*, *Sox2*, *Klf4*, and *c-Myc*. 10 days after initial transduction, GFP positive (GFP^+^) colonies could be observed (Fig. 1A). These GFP^+^ cells maintained embryonic stem cell morphology. Immunocytochemistry showed that these cells expressed typical pluripotent cell markers, including alkaline phosphatase (AP), Nanog, and SSEA1 (Fig. 1B).

Studies already demonstrate that inhibitors of histone deacetylases (HDACs), glycogen synthase kinase 3 beta (GSK-3β) or transforming growth factor beta (TGF-β) kinase contribute to efficient cell reprogramming (Li et al., 2010). Thus we investigated the potential reprogramming-promoting activity via combinations of inhibitors of these pathways. Two sets of chemical compounds inhibiting diverse pathways were examined. One set included VPA (valproic acid, HDAC inhibitor), CHIR99021 (GSK-3β inhibitor), and Repsox (TGF-β inhibitor). The other one contained NaB (sodium butyrate, HDAC inhibitor), LiCl (lithium chloride, GSK-3β inhibitor), and SD-208 (TGF-β inhibitor). Under our experimental condition, we found that both cocktails VPA/CHIR99021/Repsox and NaB/LiCl/SD-208 could achieve in the most prominent reprogramming-enhancing effects, comparing with each compound alone or pairwise combination (Figs. 1C, 1D, and S1).

### Drug cocktails robustly enhance cell reprogramming *in vitro*

**Figure 2:**
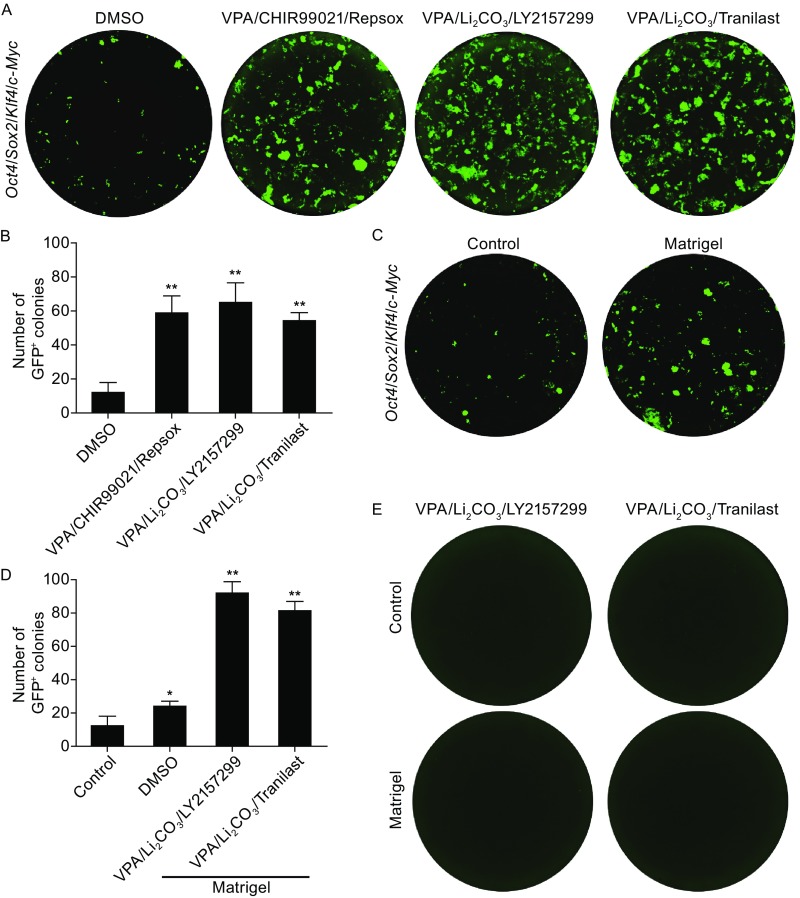
Combination of drugs promotes cell reprogramming *in vitro*. (A) Combination of chemical compounds or combination of drugs promotes GFP^+^ pluripotent stem cell generation from Yamanaka factors-induced OG2-MEFs. Images were taken on day 10 post-infection. VPA, 0.5 mmol/L. CHIR99021, 3 μmol/L. Repsox, 1 μmol/L. Li_2_CO_3_, 0.3 mmol/L. LY2157299, 1 μm/L. Tranilast, 30 μmol/L. (B) Quantification of GFP^+^ colonies in (A). (C) Generation of GFP^+^ pluripotent stem cell from Yamanaka factors-induced OG2-MEFs with or without Matrigel. Images were taken on day 10 post-infection. (D) Quantify the GFP^+^ colony numbers from Yamanaka factors-induced OG2-MEFs 10 days post-infection with diverse treatments. (E) No GFP^+^ colonies was observed 28 days after OG2-MEFs treated with combination of drugs, without introduction of Yamanaka factors. All figures are representative of three independent experiments (*n* = 3). All data are presented as mean ± SD. *P < 0.05, **P < 0.01 vs. DMSO or control

Since inhibitors of HDACs, GSK-3β, and TGF-β kinase have been applied against diseases including cancer and mental illness in clinic (Johnstone, 2002; Avila and Hernández, 2007; Ikushima and Miyazono, 2010), we further accessed the reprogramming-promoting efficiency of pharmaceutical compounds from these pathways. VPA has been used clinically against cancer (Atmaca et al., 2004), thus it is remained for ongoing test. Li_2_CO_3_ (Lithium carbonate, GSK-3β inhibitor) has been prescribed against manic and bipolar disorders (Solomon et al., 1997). Among inhibitors of TGF-β, LY2157299 has also been considered as an anti-cancer agent (Bueno et al., 2008), and Tranilast has been widely used as an antioxidant (Holmes et al., 2000), antiallergic (Kondo et al., 1992), antiangiogenesis (Isaji et al., 1997), and anti-inflammatory agent (Shiota et al., 2010). Our results showed that combination of these drugs showed robust reprogramming-enhancing effect (Fig. 2A). Reprogramming efficiency promoted by cocktail containing 0.5 mmol/L VPA, 0.3 mmol/L Li_2_CO_3_, and 1 μmol/L LY2157299 or cocktail including 0.5 mmol/L VPA, 0.3 mmol/L Li_2_CO_3_, and 30 μmol/L Tranilast was comparable to that by VPA/CHIR99021/Repsox (Fig. 2B).

Considering cell reprogramming inducted by injury *in situ* associates with microenvironment, the key factor involved is extracellular matrix (ECM). It is important to examine the effect of ECM on cell reprogramming. Matrigel containing multiple growth factors and cytokines was used to mimic ECM here. Cell reprogramming efficiency was almost twice-fold higher in Matrigel-treated group in comparison with that in control group, which could be calculated from the number of GFP^+^ colony formation (Fig. 2C). Furthermore, the reprogramming efficiency by drug cocktails was also significantly enhanced after Matrigel treatment, which is even slightly higher than that under non-Matrigel treatment (Fig. 2D). Thus drug cocktails VPA/Li_2_CO_3_/LY2157299 or VPA/Li_2_CO_3_/Tranilast could significantly improve cell reprogramming, which was even better in favor of extracellular matrix. However, none of the two drug cocktails alone could generate GFP^+^ pluripotent cells from OG2-MEFs without introduction of exogenous Yamanaka factors, no matter combining with or without Matrigel (Fig. 2E).

### Drug cocktails protects liver against acute injuries

**Figure 3:**
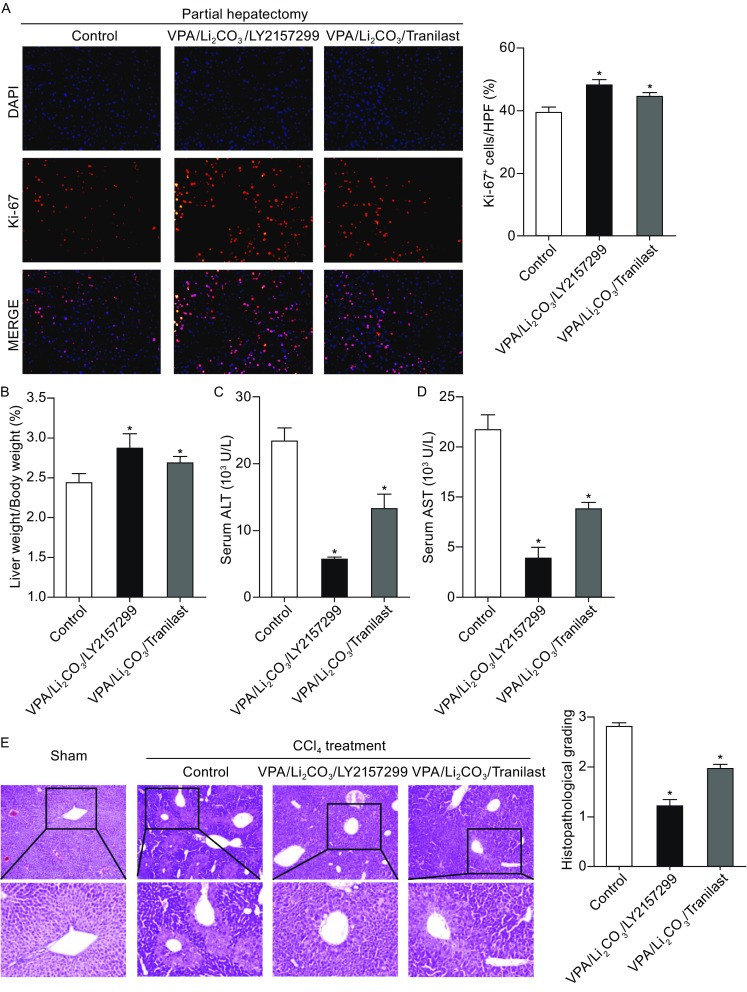
Drug cocktails protect liver from acute injuries. (A and B) Drug cocktails protect liver from acute injury induced by partial hepatectomy (PHx). (A) Representative images of Ki-67 positive cells (red) at 48 h after PHx are shown in the left panels. Nuclei were stained with DAPI. Right panels show quantification of Ki-67 positive (Ki-67^+^) cells. HPF, high-power fields. (B) Assessment of liver regeneration treated with drug cocktails after PHx by measuring ratio between liver weight and body weight. (C–E) Drug cocktails protect liver from acute injury induced by CCl_4_ treatment. (C and D) Serum alanine transaminase (ALT) and aspartate transaminase (AST) activity were analyzed 24 h after CCl_4_ treatment. (E) Representative images of the liver histopathological changes are shown in the left panels and quantification of histopathological grading is shown in the right panel. All data are presented as mean ± SD. * *P* < 0.05 vs. control

Then we tested whether administration of these drug cocktails would be helpful for tissue recovery after damage. Partial hepatectomy (PHx) is well-established model for studying liver regeneration (Mitchell and Willenbring, 2008). Drug cocktail VPA/Li_2_CO_3_/LY2157299 or VPA/Li_2_CO_3_/Tranilast was administrated intraperitoneally 6 h after PHx. Drug dosage in each cocktail was according to previously reported data. 48 h after PHx, the ratio of liver weight to body weight was calculated and liver specimens were harvested and sectioned for immunofluorescent staining of proliferation marker Ki-67 to assess liver regeneration. As shown in Fig. 3A, cells in livers that received drug cocktails showed enhanced cell proliferation. Consistently, in drug cocktail-treated mice, the ratio of liver weight to body weight evaluated significantly higher than that in saline-treated mice (Fig. 2B), indicating that both drug cocktails improved liver regeneration after physical damage.

Carbon tetrachloride (CCl_4_) has been used to induce acute or chronic liver injury and leads to liver function failure (Lindroos et al., 1991). 6 h later following CCl_4_-administration, VPA/Li_2_CO_3_/LY2157299 or VPA/Li_2_CO_3_/Tranilast was injected intraperitoneally. 24 h after CCl_4_-treatment, serum alanine transaminase (ALT) and aspartate transaminase (AST) levels were monitored for the function of liver recovery. As shown in Fig. 3C and 3D, ALT and AST levels were significantly lower in study groups treated with drug cocktails comparing with that in saline-treated control group, suggesting CCl_4_-induced acute failure or hepatic dysfunction was attenuated by the drug cocktails. Liver histopathology analysis was then performed to assess the correlated hepatocellular damage. As shown in Fig. 3E, CCl_4_-induced acute liver injury including hepatocyte ballooning and necrosis was also attenuated by the drug cocktails. However, treatment with VPA/Li_2_CO_3_, LY2157299 or Tranilast alone showed no promotional effects for liver recovery from either PHx or CCl_4_-induced acute liver injury (Fig. S2).

### Drug cocktails enhance expression of pluripotent genes

**Figure 4:**
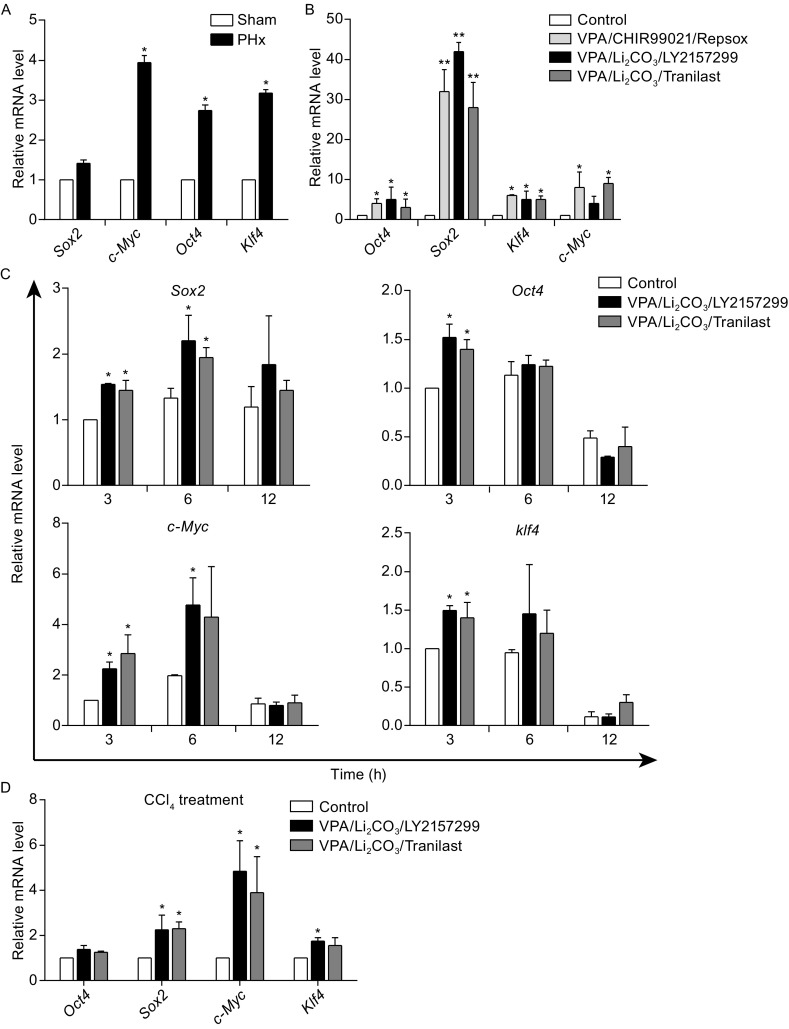
Combination of chemical compounds enhances pluripotent gene expression. (A) qRT-PCR analysis of pluripotent gene expressions in liver tissues 6 h after partial hepatectomy (PHx). (B) qRT-PCR analysis of endogenous pluripotency gene expression in OG2-MEFs 6 h after treatment with Yamanaka factors and diverse combination of chemical compounds. (C) Analyze the expression of pluripotent genes by qRT-PCR at different time points in liver tissues, after treatment with diverse drug cocktails in mouse model of liver regeneration by partial hepatectomy (PHx). (D) Analysis of pluripotent gene expression in liver tissues by qRT-PCR, 6 h after treatment with diverse drug cocktails in CCl_4_ induced acute liver injury mouse model. All data are presented as mean ± SD. *P < 0.05 vs. sham or control

Expression of pluripotent genes is up-regulated instantly in regenerative liver after acute injury, which indicated that these factors play a role during liver regeneration (Bhave et al., 2011). To verify this phenomenon, 6 h after PHx without drug treatment, liver samples were harvested for quantitative real-time PCR analysis. The result revealed that expression level of *c-Myc*, *Oct4*, and *Klf4* was significantly higher 6 h after PHx than that in sham, while expression level of *Sox2* was slightly enhanced (Fig. 4A). Since combination of small chemical compounds with diverse targets highly promote cell reprogramming *in vitro*, it is very interesting to know whether these cocktails also enhance expression of pluripotent genes in this process. 6 h after treatment of VPA/CHIR99021/Repsox, or VPA/Li_2_CO_3_/Tranilast in OG2-MEFs transduced with Yamanaka factors, expression level of *Oct4*, *Sox2*, *Klf4*, and *c-Myc* was significantly elevated comparing with no chemical treatment, especially for *Sox2*. For cocktail VPA/Li_2_CO_3_/LY2157299, it significantly activated the expression of *Oct4*, *Sox2*, and *Klf4* (Fig. 4B).

*In vivo* assays, expression levels of four pluripotent genes *Sox2*, *Oct4*, *Klf4*, and *c-Myc* were significantly elevated as early as 3 h after treatment of both drug cocktails in liver samples with PHx. The significant enhancement of *Sox2* expression lasted for 6 h in both drug treated groups. Expression of *c-Myc* was still significantly higher 6 h after treatment of VPA/Li_2_CO_3_/LY2157299. Expression levels of all these genes were reduced 12 h after PHx in liver treated with either drug cocktail or saline (Fig. 4C). For CCl_4_-induced acute liver injury, 6 h after administration of drug cocktail, expression of *Sox2*, *c-Myc*, and *Klf4* was significantly improved in VPA/Li_2_CO_3_/LY2157299 treated group, while VPA/Li_2_CO_3_/Tranilast only enhanced the expression of *Sox2* and *c-Myc* (Fig. 4D).

## Discussion

Pluripotent genes are activated in injured tissue cells and play important role in regeneration. By detecting orthologs of mammalian reprogramming factors in newts, expression of *Sox2*, *Klf4*, and *c-Myc* are upregulated during lens and limb regeneration (Maki et al., 2009). In the process of chick retinal pigmented epithelial (RPE) cell dedifferentiation and transdifferentiation after retinal injury, expression of these three pluripotent genes are also upregulated in injured RPE during short time comparing with non-injured one. Expression of these genes is further enhanced and cell reprogramming process is sustained after addition of FGF2 (Luz-Madrigal et al., 2014). In adult zebrafish, expression of *Oct4* and *Sox2* is upregulated in injured spinal cord as shown by time course analysis (Hui et al., 2015). Importantly, it is shown that chemical compound FM19G11 increased the expression of *Sox2*, *Oct4*, and *Nanog* in a dose-responsive manner, which can promote the regeneration after spinal cord injury (Rodriguez-Jimnez et al., 2012). Except for neural system, expression of the above three pluripotent genes is also detected at early time then decreased gradually in rat tracheal epithelium injured by 5-fluorouracil *in vivo*, which indicates the important role of these factors during tracheal regeneration (Song et al., 2010). During liver regeneration, *Oct4*, *c-Myc*, *Klf4*, and *Nanog* message are induced as early as 3 h after partial hepatectomy in rats. Western blot analysis demonstrates that the relative expression level of these genes is less than that in pluripotent stem cells, except for *c-Myc* (Bhave et al., 2011). Consistent with these results, our data show that expression level of pluripotent genes activated in liver can be further enhanced by cocktail of chemical compounds after acute liver injuries. Beyond the enhanced pluripotent gene expression, expression level of interleukin-6 (*Il-6*) is also significantly increased by drug cocktail treatment after PHx (Fig. S3). It has been reported that IL-6 mediates increase of cell reprogramming *in vitro* and helps enabling cellular reprogramming *in vivo* (Brady et al., 2013; Chiche et al., 2016; Mosteiro et al., 2016). Importantly, these enhancements are accompanied by improved efficiency of liver regeneration.

Transient introduction or partial activation of pluripotent genes can convert somatic cells into transition state, which can then be inducted into various cell types under specific cell culture conditions with soluble lineage-specific signals, absent of establishment of pluripotent state (Zhu et al., 2015). This strategy serves as a new avenue for generating diverse functional cell types to cell-based regeneration with more advantageous comparing with induced pluripotent stem cell technology. Many functional cells including hepatocyte (Zhu et al., 2014), cardiomyocyte (Efe et al., 2011), neural stem cells (Wang et al., 2013), and endothelial cells have been generated via this method (Margariti et al., 2012). It also has been shown that combination of complete small molecules promoting cell reprogramming can convert somatic cells into intermediate cells. These cells then directly differentiate into desirable functional cells without pluripotent state (Cheng et al., 2014; Fu et al., 2015; Tian et al., 2016). Beyond these successful *in vitro* results, it is very interesting that short-term administration of exogenous pluripotency genes through hydrodynamic tail-vein injection only reprogram hepatocytes towards pluripotency transiently without disruption of host liver function and without teratoma formation (Yilmazer et al., 2013), although long-term induction of the factors *in vivo* still can generate induced pluripotent stem cells *in situ* (Abad et al., 2013). Thus the *in vivo* cell reprogramming processes is very similar to that *in vitro*. Beyond that, the *in vivo* microenvironment can provide tissue specific factors and three dimensional context for cell survival, which may be even better than *in vitro* cell culture condition. Many desirable cells generated through *in vivo* cell reprogramming, such as cardiomyocytes derived from *in situ* cardiac fibroblasts and hematopoietic stem cells isolated from teratoma (Qian et al., 2012; Amabile et al., 2013; Suzuki et al., 2013), acquire better cell function than their counterparts from *in vitro* assays. Accordingly, here we speculate that the chemical compounds may function together with endogenous pluripotency genes that were activated instantly by liver injury and promote reprogramming of parenchymal or non-parenchymal cells into “activated cells” with more plasticity. Then these cells would adaptively re-differentiate into new desirable cells under niche conditions *in vivo*, which contribute to liver regeneration (Yanger et al., 2013; Jessen et al., 2015).

It is still a big issue for introduction of exogenous genes by virus in clinical translation due to safety concern including tumorigenesis. However, small chemical compounds are much safer and have shown more advantageous in modulating cell function and cell fate changes (Xu et al., 2008). Here, each drug in our cocktails has been approved individually for their dosage safety as well as the tolerability, pharmacokinetics and pharmacodynamics. Some of those drugs have been considered as agents against cancer. Moreover, only the drug cocktails cannot induce pluripotent cell generation *in vitro* and short term administration of these drugs *in vivo* just activates transient enhancement of the pluripotent gene expression. Besides, there is no harmful effect of these drug cocktails on other organs, such as kidney (data not shown here). Thus our study provides proof-of-concept evidence that clinical compounds identified through promoting cell reprogramming efficiency could be applied safely to facilitate tissue regeneration. During the preparation of this manuscript, an excellent work by Izpisua Belmonte and colleagues reported partial reprogramming *in vivo* not only ameliorates signs of aging but also improves regeneration of pancreas and muscle after injury (Ocampo et al., 2016). However, in this study, we only assess beneficial effects of the drug cocktails to liver tissue, whether these drug cocktails would also be applicable for other organs or anti-aging needs to be investigated in future.

## Materials and methods

### Cell culture

MEFs were isolated from E13.5 embryos heterozygous for the Oct4-GFP transgenic allele as previously described (Chen et al., 2010). Briefly, OG2 mice carried *Oct4* promoter driving GFP expression mated with 129 mice. Internal organs and gonads were removed before processing for cell isolation. Isolated MEFs in early passages (less than 3 passages) were used for further experiment and cultured in Dulbecco’s Modified Eagle’s Medium (DMEM) supplemented with 10% fetal bovine serum (FBS), 2 mmol/L L-glutamax, 0.1 mmol/L nonessential amino acids (NEAA), 100 units/mL penicillin and 100 μg/mL streptomycin.

### Generation of mouse induced pluripotent stem cells

Retrovirus expressing *Oct4*, *Sox2*, *Klf4* or *c-Myc* was produced in Plat-E cells as previously described (Chen et al., 2010). Briefly, 80% confluent Plat-E cells were transfected with retroviral vectors pMXs containing coding sequences of mouse *Oct4*, *Sox2*, *Klf4* or *c-Myc* by FugeneHD transfection reagent (Promega, Cat. No. E2311). 48 h later, supernatant containing virus was added into plates of MEFs cell culture with 4 μg/mL polybrene then spun at 2,000 rpm for 90 min to ensure infection. MEFs were infected with virus twice, one time per day. Two days after virus infection, infected MEFs were digested into single cell and seeded at 5,000 cells per well into 96-well plates pre-seeded with irradiated MEFs or pre-coated with Matrigel (BD biosciences, Cat. No. 356234). Medium was changed into KSR medium (knockout-DMEM supplemented with 15% knockout serum replacement, 2 mmol/L L-glutamax, 0.1 mmol/L NEAA, 0.1 mmol/L β-mercaptoethanol, 1000 U/mL LIF, 100 units/mL penicillin and 100 μg/mL streptomycin, N2 and Vitamin C) with or without chemical compounds. One week later, medium was replaced with mouse embryonic stem cell medium (DMEM supplemented with 15% FBS, 2 mmol/L L-glutamax, 0.1 mmol/L NEAA, 0.1 mmol/L β-mercaptoethanol 1000 U/mL leukemia inhibitory factor, 100 units/mL penicillin and 100 μg/mL streptomycin).

### Screening chemical compounds to promote cell reprogramming

Small chemical compounds were added into KSR medium 1 day after reseeding of virus infected MEFs into 96-well plate. Chemical treatment lasted for 3 days, then medium was changed without chemical compounds every two days. 10 days later from initial chemical treatment, each well of 96-well plate was photographed by Acumen eX3 microplate scanner (TTP Labtech) and the number of GFP^+^ colonies was automatically counted by ImageXpress^®^ Micro System (Molecular Devices). The small chemical compounds examined includes: Valproic acid sodium salt, Sigma Aldrich Cat. No. P4543, 3 mol/L stored in H_2_O. CHIR99021, Tocris Cat. No. 4423, 10 mmol/L stored in DMSO. Repsox, Selleckchem Cat. No. S7223, 10 mmol/L stored in DMSO. Sodium butyrate, Cat. No. ARK2161, 1 mol/L stored in H_2_O. Lithium chloride, Sigma Aldrich Cat. No. 746460, 5 mol/L stored in H_2_O. SD-208, Sigma Aldrich Cat. No. S7071, 10 mmol/L stored in DMSO. Lithium carbonate, Sigma Aldrich Cat. No. 62470, 2 mol/L stored in H_2_O. LY2157299, Selleckchem, Cat. No. S2230, 10 mmol/L stored in DMSO. Tranilast, Selleckchem Cat. No. S1439, 100 mmol/L stored in DMSO.

### Mouse models of acute liver injury

Male C57BL/6 mice, 10–12 weeks old, were purchased from Shanghai Laboratory Animal Center (Shanghai, China) and maintained in pathogen-free condition. 2/3 partial hepatectomy (PHx) was performed according to previous published protocol (Mitchell and Willenbring, 2008). Briefly, mice were anesthetized with pentobarbital sodium. After restraining the mouse and exposing the xiphoid, the left lateral lobe and median lobe were tied using 4-0 silk surgical thread, then the lobes were removed surgically. Sutured the abdomen and waited for the mice recovering. 6 h after operation, drug cocktail containing diverse chemical compounds as indicated was injected intraperitoneally (i.p.) into the mice. Dosage for each compounds in cocktails referenced from reported data is as follows: 100 mg/kg VPA (Hunan Xiangzhong Pharmaceutical Co., Ltd), 60 mg/kg Li_2_CO_3_ (Yangzhou Pharmaceutical Co., Ltd.), 30 mg/kg LY2157299 (Synthesized by BioChemPartner), and 25 mg/kg Tranilast (CPU-Pharma). Injection of saline is considered as control. VPA/Li_2_CO_3_/LY2157299 treated group contained 12 mice. VPA/Li_2_CO_3_/Tranilast treated group contained 12 mice. Control group contained 10 mice. 48 h after PHx, mice were sacrificed for evaluation.

Fulminant hepatic failure was induced by i.p. injection of CCl_4_ (5% *v*/*v* CCl_4_ in olive oil; 10 mL/kg body weight). 6 h after injection, mice were administrated i.p. with drug cocktails (Combination and dosage of each compound was same as the above treatment) or saline. Each group contained 12 mice. 24 h after initial CCl_4_ treatment, mice were anesthetized with sodium pentobarbital (50 mg/kg) (Sigma) by i.p. injection. Blood samples were harvested from mouse orbital sinus by capillary tube then spun down at 12,000 rpm for serum collection. Serum samples were sent to clinical laboratory of Eastern Hepatobiliary Surgery Hospital for ALT and AST analysis. Liver samples were fixed and embedded in paraffin then sectioned for immunohistochemical analysis.

### Immunohistochemistry

For induced pluripotent stem cell characterization, GFP^+^ embryonic stem cell-like colonies were picked up and passaged on irradiated MEFs in mouse embryonic stem cell medium. The 5th passage GFP^+^ colonies were fixed in 4% paraformaldehyde (PFA) and stained for alkaline phosphatase (AP) activity using AP detection kit (Sigma Aldrich, Cat. No. 85L3R). For immunofluorescent staining, fixed cells were incubated with primary antibodies against SSEA1 (Santa Cruz Biotechnology, Cat. No. sc-21702, 1:500 dilution) or Nanog (Santa Cruz Biotechnology, Cat. No. sc-293121, 1:500 dilution) overnight at 4°C after fixation, followed by secondary antibody goat anti-mouse Alexa Fluor Plus 555 (ThermoFisher, Cat. No. A32727, 1:1000 dilution) at room temperature (RT) for 2 h. Images were taken under a fluorescent microscope (Zeiss Axio Observer Z1).

Mice went through PHx were anesthetized with sodium pentobarbital and perfused with saline. Liver tissues were harvested and fixed in 4% PFA overnight then in 30% sucrose solution for 24 h. Mounted the tissues in O.C.T. embedding compound and froze at −80°C. Frozen tissues then were dissected into 10 μm thick sections. After blocking the liver sections with 0.5% bovine serum albumin in 1× phosphate buffered saline for 30 min, incubated the sections with primary antibody Ki-67 (Santa Cruz Biotechnology, Cat. No. sc-7846, 1:500 dilution) at 4°C overnight then with donkey anti-goat secondary antibody Alexa Fluor 568 (ThermoFisher, Cat. No. A-11057, 1:1000 dilution) at RT for 2 h. Images were taken under a fluorescent microscope (Zeiss Axio Observer Z1) and Ki-67 positive cells were automatically counted and analyzed by software ImageJ.

### H&E staining and histopathological analysis

For histological analysis, liver tissue specimens from CCl_4_-injuried mice were fixed in 4% PFA overnight, then dehydrated by graded ethanol at RT and embedded in paraffin. Tissue sections at 5 μm were deparaffinized by xylene and stained with hematoxylin and eosin (H&E). Histopathological analysis of liver was performed according to previous reports (Feng et al., 2010). The grades of liver damage in different groups were assigned in numerical scores (scale from 0 to 3).

### Quantitative real-time PCR

Total RNAs were extracted from cells or liver tissues using TRI Reagent^®^ (Sigma Aldrich, Cat. No. T9424) according to the manufacture’s protocols. To obtain cDNA, RNA was reverse-transcribed by M-MLV reverse transcriptase and random hexamers. cDNA, 2× PCR Mix and Eva Green were mixed and analyzed with MX3000P Stratagene PCR machine. The relative mRNA expression values were normalized against the inner control (*Hprt*). Primer sequences are listed in Table S1.

### Statistical analysis

Unless otherwise indicated, all experiments were repeated three times and all data were presented as mean ± standard deviation (SD) and analyzed using Student’s *t*-tests. A value of *P* < 0.05 was considered statistically significant. The following significance values are noted throughout the text: *P < 0.05; **P < 0.01.

## Electronic supplementary material

Below is the link to the electronic supplementary material.

Supplementary material 1 (PDF 640 kb)
