# Supplementary material for: Cocktail of chemical compounds robustly promoting cell reprogramming protects liver against acute injury
Source: Protein Cell. 2017 Feb 11;8(4):273–83. doi: 10.1007/s13238-017-0373-y (PMC5359186; doi:10.1007/s13238-017-0373-y)
Supplement: Supplementary file 1 — Supplementary material 1 (PDF 640 kb) [file 13238_2017_373_MOESM1_ESM.pdf]

Supplementary data

Cocktail of Chemical Compounds Robustly Promoting Cell Reprogramming Protects Liver against Acute Injury

Yuewen Tang, Lin Cheng

Supplementary Figure 1

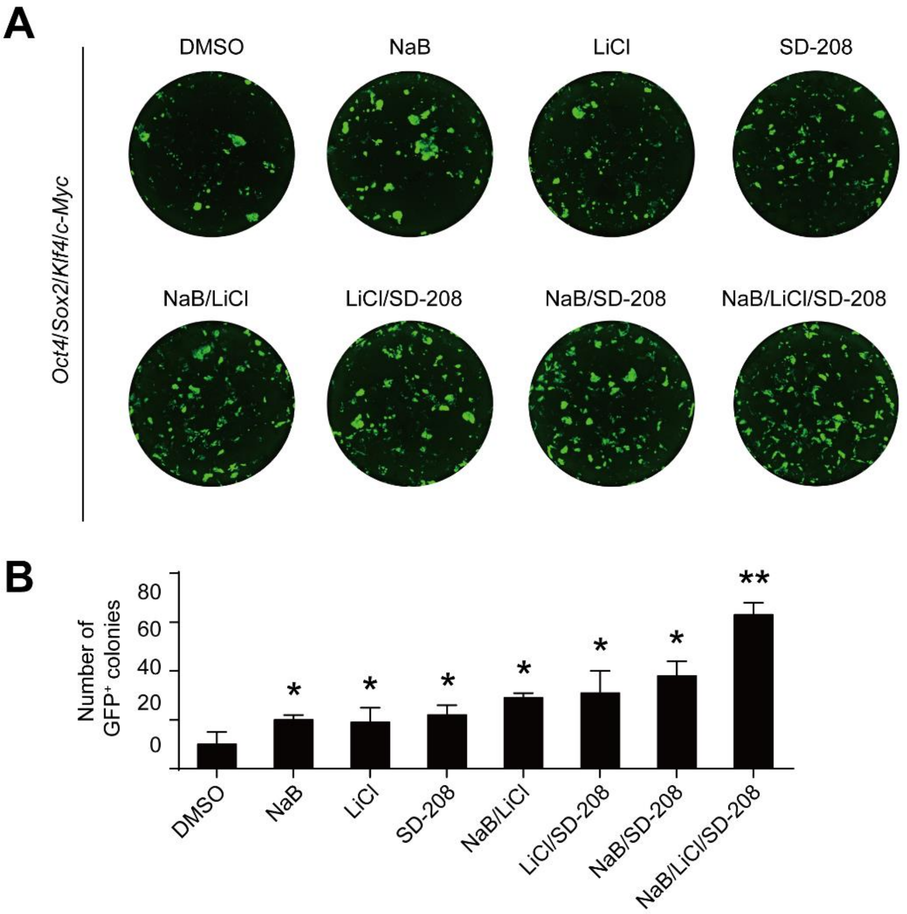

Supplementary Figure 1. Chemical compounds promote cell reprogramming *in vitro.*

(A) Generation of pluripotent stem cell from Yamanaka factors-induced OG2-MEFs under treatment of diverse chemical compounds. Images of GFP^+ colonies were taken on day 10 post-infection. NaB, 0.5 mmol/L. LiCl, 1 mmol/L. SD-208, 1 μmol/L. (B) Quantification of GFP^+ colonies in (A). All figures are representative of three independent experiments (n = 3). All data are presented as mean ± SD. *P<0.05, **P<0.01 vs. DMSO.

Supplementary Figure 2

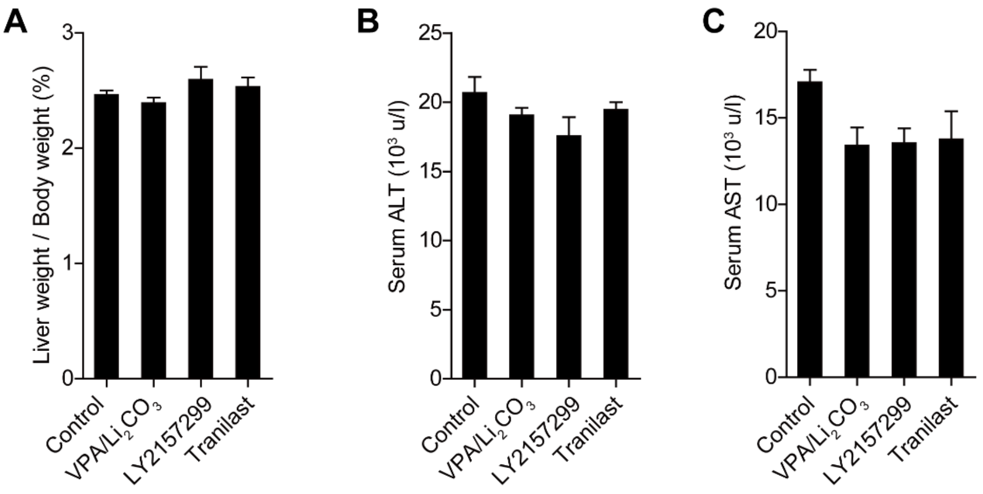

Supplementary Figure 2. Protecting effect of chemical compounds on acute liver injuries.

(A) Assessment of liver regeneration treated with diverse chemicals as indicated 48 hours after PHx by measuring ratio between liver weight and body weight. (B and C) Serum alanine transaminase (ALT) activity and aspartate transaminase (AST) activity were analyzed 24 hours post CCl_4 treatment. All data are presented as mean ± SD.

Supplementary Figure 3

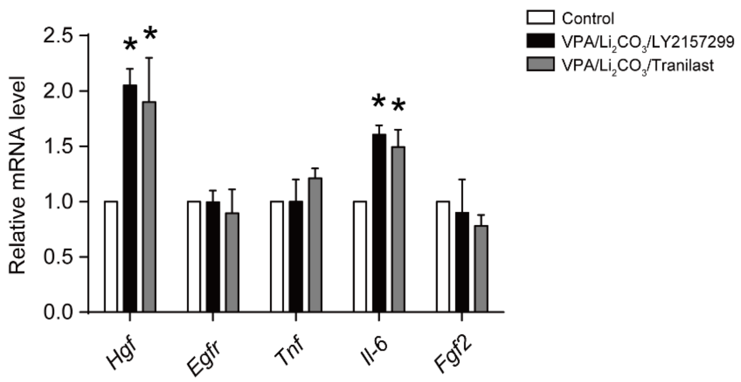

Supplementary Figure 3. Expression level of the genes playing important roles in liver regeneration was analyzed by qRT-PCR, 6 hours after treatment with diverse drug cocktails in mouse model of partial hepatectomy. Hepatocyte growth factor (*Hgf*), epidermal growth factor receptor (*Egfr*), tumor necrosis factor (*Tnf)*, interleukin-6 (*Il-6*) and fibroblast growth factor 2 (*Fgf2*). All data are presented as mean ± SD. *P<0.05 vs. control.

Supplementary Table 1. Primer sets for qRT-PCR

Mouse gene	Sequence (5'→3')	
Oct4	Forward	TCTTTCCACCAGGCCCCCGGCTC
	Reverse	TGCGGGCGGACATGGGGAGATCC
Sox2	Forward	CTGGACTGCGAACTGGAGAAG
	Reverse	TTTGCACCCCCTCCCAATTC
Klf4	Forward	TTGCGGTAGTGCCTGGTCAGTT
	Reverse	CTATGCAGGCTGTGGCAAAACC
c-Myc	Forward	GGAGTGGTTCAGGATTGGGG
	Reverse	GGGTAGCTTACCAGAGTCGC
Hgf	Forward	GGTTACAGGGGAACCAGCAA
	Reverse	ATTCCCCGTGTAGCACCAAG
Egfr	Forward	TCACCCAACTGGGCACTTTT
	Reverse	ACGACAGCGATGGGAACATT
Tnf	Forward	TGTCTACTCCTCAGAGCCCC
	Reverse	GACCCGTAGGGCGATTACAG
Il-6	Forward	CCAGTTGCCTTCTTGGGACT
	Reverse	GTCTCCTCTCCGGACTTGTG
Fgf2	Forward	CGTTGTACACTCAAGGGGCT
	Reverse	GTCCCGTTTTGGATCCGAGT
Hprt	Forward	AGTCCCAGCGTCGTGATTAG
	Reverse	TTTCCAAATCCTCGGCATAATGA
